# Knowledge of mental health legislation in Ghana: a case of the use of certificate of urgency in mental health care

**DOI:** 10.1186/s13033-018-0215-1

**Published:** 2018-06-28

**Authors:** Reindolf Anokye, Enoch Acheampong, Naomi Gyamfi, Amy Budu-Ainooson, Ernest Appiah Kyei

**Affiliations:** 1 Centre for Disability and Rehabilitation Studies, Department of Community Health, Kwame Nkrumah University of Science and Technology, Kumasi, Ghana; 2 Department of Health Education and Promotion, School of Public Health, Kwame Nkrumah University of Science and Technology, Kumasi, Ghana

**Keywords:** Health workers, Knowledge, Mental health legislation, Certificate of urgency

## Abstract

**Introduction:**

Mental illness can affect anyone irrespective of race, gender or personal characteristics. The study sought to investigate health workers’ Knowledge on Mental Health Legislation in Ghana focusing on the Certificate of Urgency.

**Methods:**

A descriptive study design was employed for this study. The study population included medical doctors, physician assistants, and nurses/midwives. A simple random sampling technique was used to select 384 respondents for the study. Data was collected through the use of semi-structured questionnaires.

**Results:**

Respondents who were psychiatrists were 9.56 times more knowledgeable in the use of Certificate of Urgency than those in other specialties like primary care, obstetrics and gynaecology, surgery and internal medicine; adjusted odds ratio (AOR) = 9.56 [95% confidence interval (CI) 1.57–65.2]. Respondents who had used the Certificate of Urgency before had 4.7 times more knowledge as compared to those who had not used it at all; adjusted odds ratio (AOR) = 4.77 [95% confidence interval (CI) 1.021–14.01].

**Conclusion:**

Knowledge of Certificate of Urgency was generally low. Authorities of the various hospitals should organize regular in-service training to enlighten all healthcare workers on the legislation governing mental healthcare in Ghana.

## Introduction

Mental illness can affect anyone irrespective of race, gender or personal characteristics [1]. It is estimated that one in four persons worldwide is classified at some point in their life as persons with mental illness [2]. In the US, nearly 46% of the total population at some point in time qualify to be classified as persons with mental illness [3]. In Ghana, WHO estimates that approximately 13% of Ghanaians suffer from a mental disorder: of those, 3% suffer from a severe mental disorder and the other 10% suffer from a moderate to mild mental disorder [4].

Many countries have passed Mental Health Acts. Examples include Sweden (Act 2003) in October 2005 and India (Act, 1987) effected in 1993 which replaced the Indian Lunacy Act, 1912. In Ghana, the recent Mental Health Act (MHA) was passed in 2012. The Mental Health Act (MHA) seeks to protect persons with mental illness [5]. MHA is useful and covers not only the rights of patients and how to safeguard or protect individual rights but also include situations where one can be taken to a hospital against his or her will and occasions when one can be treated against his or her will [5].

One area that the MHA sought to encourage is the use of Certificate of Urgency [6]. In mental health care, the Certificate of Urgency is used for the involuntary treatment of persons with mental illness [7].

Even though there are legislative instruments that cater for persons with mental illness which are in the form of Mental Health Acts, there is continuous discrimination and stigma against persons with mental illness [8, 9]. Also, many countries do not consider mental health care as a vital component of public health care [10].

According to Ghana’s Mental Health Act (846) Section 48, clause 2 and 3, when a registered medical practitioner examines a person with possible diagnosis of mental illness and meets the criteria for emergency care, the medical practitioner then issues a Certificate of Urgency to place the person under care, observation, and treatment. Also, any healthcare professional irrespective of their specialty may seek the assistance of the police to take a person to a health facility in a situation where the person is suspected to be suffering from a mental disorder for a Certificate of Urgency to be issued after examination by a medical practitioner.

Anecdotal evidence suggests that despite the provision of the Mental Health Act of Ghana, there seems to be less publicity about the use of the Certificate of Urgency as many citizens and even some health officers seem not to be aware of its existence.

In a study undertaken in Scotland which examined psychiatrists’ knowledge of mental health legislation. The study findings revealed that knowledge of most basic definitions and fundamental areas was limited [11]. A study that was carried out to determine the levels of knowledge of Section 136, The Mental Health Act 1983 in the UK by Lynch et al. [12] also found a majority of respondents having low knowledge of the Act 1983. Passmore and Leung [13] study among psychiatrists found that half of the respondents did not know what certain provisions of the Human Rights Act stipulate. In Lovell et al. [14] study conducted among nurses holding powers in England, only 45% of the sample (164 nurses) knew the criteria set out in the Mental Health Act Code of Practice. Houlihan [15] study found that nurses’ required more training on treatment without consent under the Mental Health Act 1983 in England.

Knowledge levels of mental health legislation among health workers’ in Ghana is scanty. Therefore, the study sought to investigate health workers’ knowledge on mental health legislation in Ghana focusing on the Certificate of Urgency. The study will bring to bear the extent to which healthcare workers are knowledgeable when it comes to the use of the Certificate of Urgency. This will enable stakeholders to assess the knowledge base of health officials in mental healthcare in order to plan ways to improve it. The study will expose the use of Certificate of Urgency in Ghana. This will enable readers to become fully aware of the functionality of the Certificate of Urgency and enlighten them on their role in improving mental healthcare.

## Methods

A descriptive design was employed in this study and the design was selected based on the fact that descriptive research allows the researcher to only express what is happening on the field without any additions from the researcher’s perspective. The researcher’s duty in descriptive research is to only observe and present exactly the phenomenon of study [16]. Specifically, the health officer’s use of Certificate of Urgency in mental healthcare was expressed as it existed on the field devoid of the investigator’s opinion and sentiments.

The targeted population for the study was Medical Doctors, Physician Assistants, and Nurses who as part of their duties are engaged in providing treatment and referral services to persons with mental illness. The study population comprised of all health officials of 7 public hospitals in 3 regions (Central, Greater Accra, and Ashanti) of Ghana.

A multi-stage sampling technique was employed where three (3) regions were purposively selected due to the reason that the three psychiatric hospitals in the country are located in 2 of the regions (Central and Greater Accra) and the other being Ashanti has one of the largest hospitals in Ghana. After that, seven (7) hospitals were also purposively selected based on the availability of psychiatrists or mental health practitioners (Registered mental nurses, community mental health officers, and psychiatrists). These hospitals were selected in order to increase the probability of selecting a significant number of psychiatrists or mental health practitioners among other health workers. A simple random sampling technique was then used to select the respondents from the hospitals. The probability sampling was used in order to also include other health professionals who may not be psychiatrist but play other roles when it comes to mental health as in the case of midwives who play a significant role in the management of postpartum depression/postpartum psychosis.

Respondents were randomly selected by letting them pick a paper that was folded and had ‘Yes’ or ‘No’ written in it. Those who picked ‘Yes’ were selected till the minimum sample size was achieved. In this study, the minimum sample size was estimated using the formula of Cochran [17] which is recommended for studies where the total population is not known.

The formula is;

$$n_{0} = \frac{{z^{2} \times p\left( {1 - p} \right)}}{{e^{2} }}.$$

The following values were used for estimating the sample size—*n*_0_: ?, *z*^2^: 95% confidence level (The value of (1 − α) in standard normal distribution *z*-table, which is 1.96 for 95%), *p*: 50% variability of the population (which is maximum), *e*: 5% margin of error. Where, *n*_0_: sample size, which was estimated, *z*^2^: selected critical value of the desired level of confidence or risk, *p*: estimated proportion of an attribute that is present in the population or maximum variability of the population, *e*: the desired level of precision or margin of error.

The values were placed in the formula;

$$n_{0} = \frac{{\left( {1.96} \right)^{2} \times 0.5\left( {1 - 0.5} \right)}}{{\left( {0.05} \right)^{2} }} = 384.16.$$

The minimum sample size of 384 was obtained. Therefore, a sample of 384 was selected for the study. However, out of the estimated sample of 384 respondents, 5 exited at the early stages of the study and 379 respondents completed the study. Therefore the margin of error was 0.58%.

Data was collected through the use of semi-structured questionnaires and were self-administered by respondents. A pre-test was conducted in two different regions to ascertain the potency of the sampling procedure and the viability of the research instruments. Cronbach’s Alpha was used to test the viability of the questionnaires and the value of the Cronbach’s Alpha was 0.62 (more than 0.5 and above are recommended values), the questionnaire was subsequently modified to achieve higher viability. This made the instrument more appropriate which improved data collection. Difficulties in interpreting information in the research instruments were corrected by simplifying the wording.

The investigators personally arranged for the administration  of the research instruments after a prior visit that assisted in refining timings of distribution of questionnaires. The investigators agreed with the respondents when the research instruments were to be administered and the specific date of collecting the questionnaires. A period of one (1) week was allowed for the respondents to answer the questionnaire.

To render the study ethical, the right of self-determination, anonymity, confidentiality and informed consent were observed. Written and verbal permission was obtained to conduct the study in the hospital. Respondents were told the purpose of the study, the procedure that would be used to collect the data, and assured them that there were no potential risks or cost involved. Scientific sincerity is regarded as a very important ethical responsibility when conducting research. Therefore the researcher took the following ethical consideration in the process of collecting the data for the study. The respondents for the study were  allowed to participate voluntarily in the research without been persuaded. The reason for the observance of this was that if they are not allowed to participate out of their own volition, they will give false information that will mar the objectives of the research. The respondents of the study were told the objectives of the study, the possible implication and effect of the research. As a result of this, the information given was based purely on informed consent. The confidentiality of the study was paramount and observed by the researcher. The data collected were managed and used in such a way that the identity of the respondents were  protected and that no information can be directly traced or associated with any individual respondent. All references were duly acknowledged to prevent plagiarism.

Data cleaning was done which involved identification of incomplete or inaccurate responses and completed ones. After data cleaning, the data were coded and entered into the computer for analysis using the Statistical Package for Social Sciences (SPSS) version 20 and the excel spreadsheet. Results have been presented in percentages and frequencies through frequency tables and pie charts.

## Results

### Demographic characteristics of respondents

Table 1 indicates the demographic characteristics of the respondents for the study. The demographic variables of respondents studied covered age, gender, professional qualification, specialties and  years of working experience. More than half of the respondents were females constituting 53%. The highest age group among the respondents was between the age range 26–35 years. Out of the total number of respondents, 88% were nurses/midwives representing the largest professional group. With regard to the specialties of the respondents, 52% of respondents indicated primary care as their main specialty field while 4% of the respondents practiced psychiatry/mental healthcare. Over half (53%) of the total number of respondents had between 1 and 5 years working experience with only 3% having 16 years and more working experience.

**Table 1:** Demographic characteristic of respondents

Demographic variables	Frequency (n = 379)	Percentage (%)
Gender		
Female	202	53
Male	177	47
Age		
18–25	122	32
26–35	173	46
36–45	65	17
46–55	10	3
56 and above	9	2
Professional qualification		
Med. doctor	16	4
Phy. assistants	29	8
Nurse/midwife	334	88
Specialties		
Primary care	198	52
Obstetrics and gynecology	96	25
Surgery	22	6
Psychiatry/mental health	16	4
Internal medicine	47	12
Years of work (years)		
1–5	213	56
6–10	121	32
11–15	34	9
16 years and above	11	3

### Knowledge of Certificate of Urgency

The knowledge of respondents on Certificate of Urgency was assessed to indicate their level of awareness on specific regulations guiding the use of Certificate of Urgency. From Table 2, close to half (47%) of respondents were aware of the existence of Certificate of Urgency under the Mental Health Act. Less than half (42%) of the total number of respondents indicated that a patient under Certificate of Urgency can be detained not exceeding 72 h. Respondents who were aware of the specific regulation that “a person shall be received and detained in any other place of safe custody for a period not exceeding 48 h” were 127 representing 34% of the total number of respondents. Regarding their awareness of the existence of any procedure in issuing the Certificate of Urgency, the majority (56%) indicated their awareness of existing proceedings in the issue of the Certificate of Urgency. Among the places where Certificate of Urgency could be obtained, Ghana Health Service were the commonest place mentioned by  32% of the respondents who were aware that Certificate of Urgency could be obtained from that end whilst 26% of the respondents were not aware of where the Certificate of Urgency could be obtained.

**Table 2:** Knowledge of Certificate of Urgency

Variables	Frequency (n = 379) %
Awareness of Certificate of Urgency under mental health act	
Yes	179 (47%)
No	200 (53%)
A patient under the Certificate of Urgency may be detained not exceeding 72 h	
Yes	219 (58%)
No	160 (42%)
A person shall be received and detained in any other place of safe custody for a period not exceeding 48 h	
Yes	127 (34%)
No	255 (66%)
The existence of any procedure in issuing the Certificate of Urgency	
Yes	213 (56%)
No	166 (44%)
Where the Certificate of Urgency can be obtained	
Any health facility	41 (11%)
Ministry of health office	69 (18%)
Ghana health service officers	120 (32%)
Psychiatric hospital	49 (13%)
Don’t know	100 (26%)

### Use of Certificate of Urgency

**Fig. 1:**
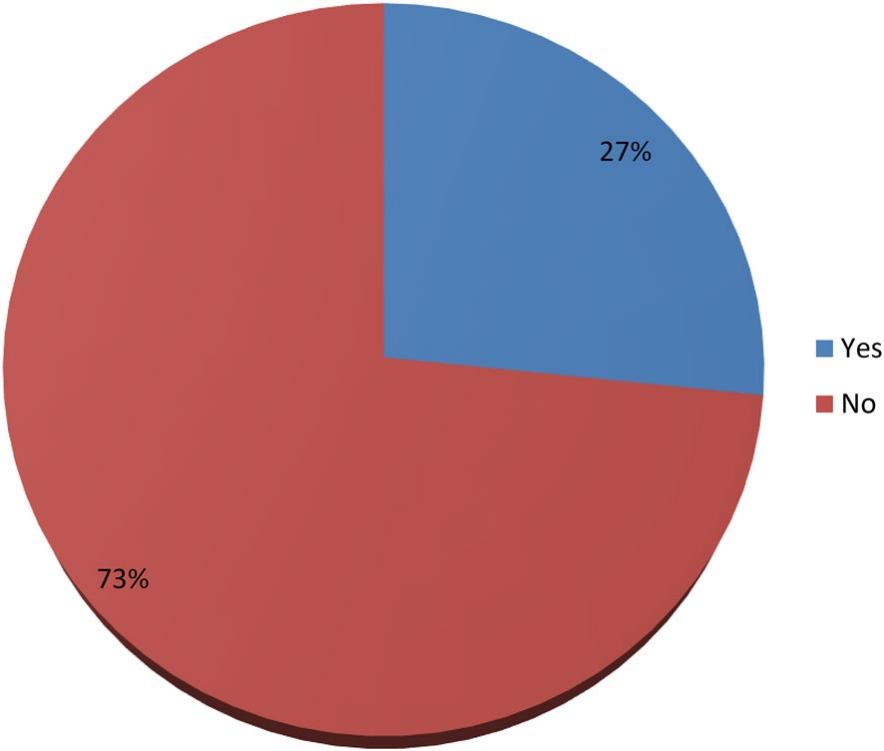
Use of Certificate of Urgency

Figure 1 illustrates respondents’ utilization of Certificate of Urgency in their field of practice. The majority (73%) of the respondents had never used Certificate of Urgency in their field of practice with less than half (27%) of the respondents indicating the usage of Certificate of Urgency.

### Instance the hospital can issue the Certificate of Urgency

**Fig. 2:**
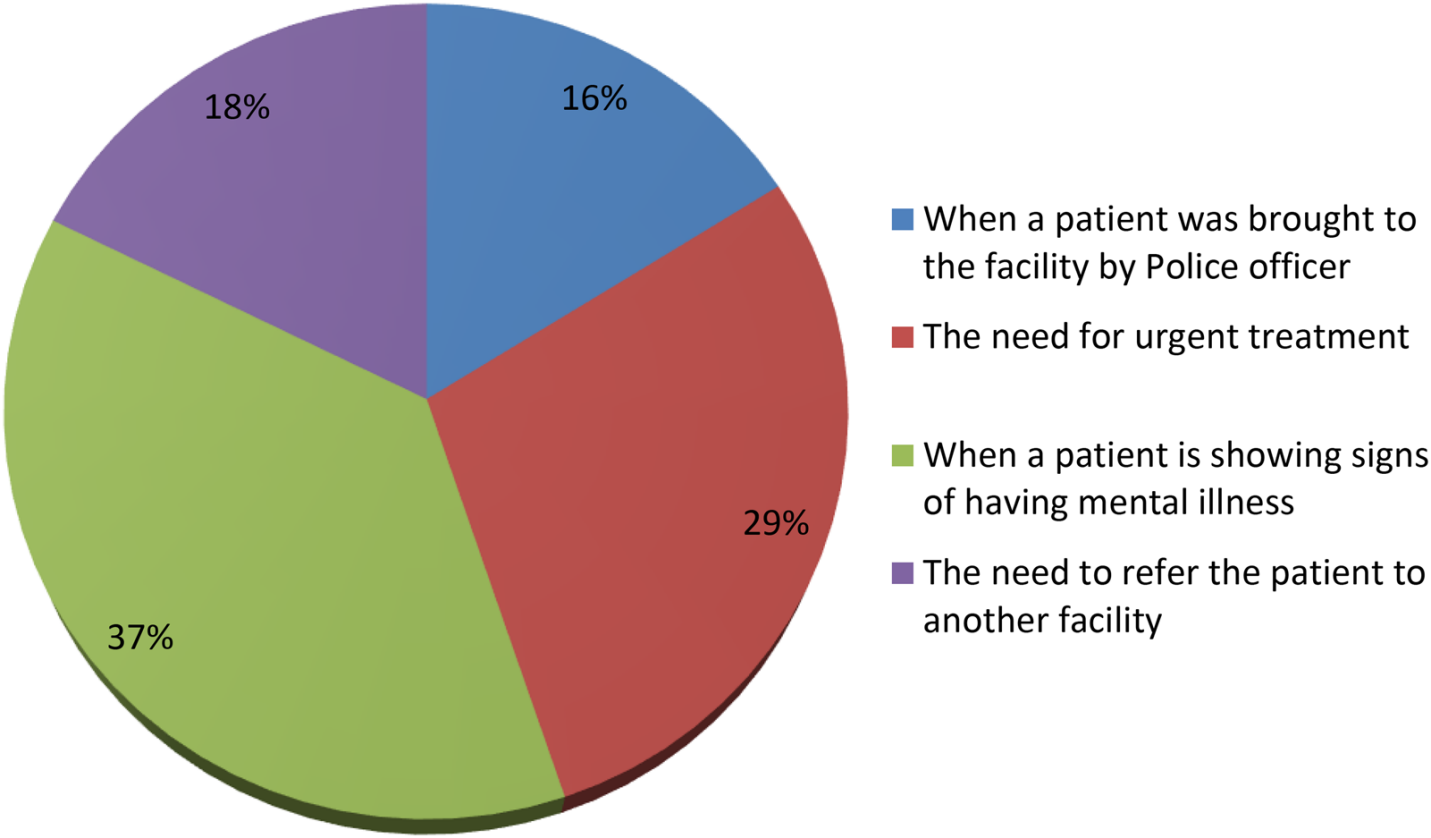
Instance the hospital can issue the Certificate of Urgency

From Fig. 2, it can be observed that 37% of the respondents stated that a Certificate of Urgency can be issued when a patient is showing signs of having a mental illness. Other notable cases mentioned by respondents where Certificate of Urgency could be used includes when there is the need for urgent treatment (29%), the need to refer the patient to another facility (18%) and when a patient is brought to the facility by police officer (16%).

### Association between demographic characteristics, the use of Certificate of Urgency and Knowledge of Certificate of Urgency

Table 3 summarizes the result of the univariate and multivariate analysis of the association between demographic characteristics as well as the use of Certificate of Urgency and knowledge of Certificate of Urgency. In both the univariate analysis and the multivariate analysis, specialty of the health workers and the use of the Certificate of Urgency had an association with having adequate knowledge of the Certificate of Urgency. Respondents who were psychiatrists were 9.56 times more knowledgeable when it comes to issuance and other information related to the  Certificate of Urgency than those in other specialties like primary care, obstetrics and gynaecology, surgery and internal medicine; adjusted odds ratio (AOR) = 9.56 [95% confidence interval (CI) 1.57–65.2]. Respondents who had used the Certificate of Urgency before had 4.7 times more knowledge as compared to those who had not used it at all; adjusted odds ratio (AOR) = 4.77 [95% confidence interval (CI) 1.021–14.01].

**Table 3:** Odds ratio with 95% confidence interval for association between demographic characteristics as well as the use of Certificate of Urgency and Knowledge of Certificate of Urgency

Variables	Knowledge of Certificate of Urgency		Univariate		Multivariate^a^	
	Adequate (n)	Inadequate (n)	OR (95% CI)	P-value	AOR (95% CI)	P-value
Gender						
Female	72	130	1.10 (0.48–2.48)	0.797	0.85 (0.19–3.09)	0.698
Male	68	109	1.00		1.00	
Professional qualification						
Med. doctor	14	2	1.02 (0.44–2.35)	0.838	1.62 (0.41–5.66)	0.529
Phy. assistance	15	14	1.00		1.00	
Nurse	111	223	1.14 (0.32–4.01)	0.912	2.00 (0.17–25.05)	0.636
Specialties						
Primary care	68	130	1.00		1.00	
Obst. and gynecol.	50	46	2.01	0.139	1.21 (0.28–6.27)	0.706
Surgery	6	16	1.00			
Psychiatry	12	4	12.5 (3.54–45.49)	< 0.001	9.56 (1.57–65.2)	0.014
Internal medicine	4	43	6.33 (0.08–1.56)	0.112	0.44 (0.08–2.46)	0.255
Years of work (years)						
1–5	73	140	1.00		1.00	
6–10	42	79	0.89 (0.41–3.02)	0.797	1.29 (0.35–4.81)	0.122
11–15	22	12	2.86 (1.13–20.28)	0.121	1.31 (0.04–2.19)	0.211
16 years and above	3	8	1.00		1.00	
Use of the Certificate of Urgency						
Yes	81	20	8.21 (3.12–20.18)	< 0.001	4.77 (1.021–14.01)	0.044
No	59	219	1.00		1.00	

*OR* odds ratio, *CI* confidence interval, *AOR* adjusted odds ratio

^a^ Mutually adjusted

## Discussions

A study by Humphreys [11] to assess psychiatrists’ knowledge of mental health legislation indicated inadequate knowledge on the foundational and essential areas within the mental health legislation. A similar finding was evidenced in this study as health workers were not adequately aware of certain aspects of the Mental Health Act and specific regulations guiding the issuance and usage of Certificate of Urgency. This may be as a result of the lack of training of health professionals in mental health legislation in the provision of mental healthcare. Respondents who were psychiatrists had 9 times more adequate knowledge when it comes to the issuance and related information on  Certificate of Urgency than those in other specialties like primary care, obstetrics and gynaecology, surgery and internal medicine. It is important for the authorities to increase their efforts in organizing training programmes to educate and enlighten all health workers within their healthcare facilities on mental health legislation in the provision of mental healthcare. This has the potential of increasing the knowledge of healthcare providers on mental health issues which will eventually improve their performance. This is important because it has been reported in Nigeria that the provision of in-service training through the development of strategies for the promotion, prevention, management, treatment and rehabilitation of mental and neurological disorders has improved the performance of healthcare professionals when it comes to management of mental illness [18].

Another study undertaken by Lynch et al. [12] on the levels of knowledge of Section 136 of the Mental Health Act 1983 in the UK found different results compared to what was found  in this study. In Lynch et al. [12] study, 179 questionnaires were administered to 30 senior doctors, 24 senior house officers, 33 senior nurses and 92 police officers. Out of these respondents, 10% of the health workers who were Accident and Emergency practitioners and 23% of the police had been formally trained on the Mental Health Act 1983. The discrepancies between these two studies could arise from the nature of participants and health workers specialties included in the various studies. In this study, police officers were not included and participants had no specialty of accident and emergency staff. A further investigation could be done to assess the knowledge of police officers who may have the power to hold up suspected persons with mental illness in public places and keep them safe.

The Mental Health Act of Ghana sets out established guidelines for the treatment of mental disorders and in the issuing of the Certificate of Urgency. The study found that more than half of the respondents (56%) knew the existing procedures in issuing the Certificate of Urgency. However, less than half of the sample (45%) of nurses in England in a study conducted by Lovell et al. [14]. was cognizant of the specific principles set out in the Mental Health Act Code of Practice. This may be as a result of the inclusion of other health professionals in this study who may be conversant with the procedure in issuing the Certificate of Urgency as compared to the use of the only nurse in the study in England.

### Strengths of the study

The study has provided vital information on the Mental Health Act that will support mental healthcare in Ghana. The sample size was large enough and healthcare professionals from different specialties were included in the study.

### Limitations of the study

The scope of the study was limited to southern Ghana, therefore, ignoring the other mental health practitioners and health workers in the northern part of the country. The Procedure for the issuance of a Certificate of Urgency also involves police officers and family members of persons with mental illness’. However, they were not included in the study due to the focus on health workers. Whilst there may be limitations inherent in the study and approaches used, these by no means compromise the results reported.

## Conclusion

The knowledge of respondents on Certificate of Urgency was assessed based on the findings, it can be concluded that knowledge on the Certificate of Urgency and its utilization among health workers was generally low. Health workers in psychiatry, however, were more knowledgeable when it comes to the use of Certificate of Urgency than those in other specialties as they have received specific training on mental healthcare.

## Recommendation

It is recommended that the authorities of the various hospitals should organize regular in-service training programmes, refresher courses and workshops to educate and enlighten all health workers within their healthcare facilities on mental health legislation in the provision of mental healthcare.

Again, the Ministry of Education should collaborate with the Ghana Health Service to revise the curriculum of the various health training institutions across the country to  enhance training on mental health legislation.

## Abbreviations

MHA: Mental Health Act
AOR: adjusted odds ratio
OR: odds ratio
CI: confidence interval
SPSS: Statistical Package for Social Sciences
